# Optimization of water and nitrogen management strategies for jujube under sand tube irrigation based on regression analysis and TOPSIS method

**DOI:** 10.3389/fpls.2026.1859716

**Published:** 2026-07-15

**Authors:** Youshuai Bai, Fang Wang, Chengbao Wang, Xiaogang Liu, Lin Huo, Xia Zhao, Xiangyi Fang, Shenghai Jia

**Affiliations:** 1 Institute of Soil, Fertilizer and Water-Saving Agriculture, Gansu Academy of Agricultural Sciences, Lanzhou, China; 2 Dingxi City Agricultural Product Quality and Safety Monitoring and Testing Station, Dingxi, China; 3 College of Water Conservancy and Hydropower Engineering, Gansu Agricultural University, Lanzhou, China; 4 Qinfeng Forestry Experimental Station of Minqin County, Wuwei, China

**Keywords:** jujube quality, jujube yield, management strategies, sand tube irrigation, TOPSIS method

## Abstract

**Introduction:**

Sand tube irrigation (STI) is one of the most considerable irrigation techniques for increasing water productivity in arid areas. However, it is unclear whether there is still room for further improvements in jujube yield and water productivity under STI with different irrigation and nitrogen application levels.

**Methods:**

To solve this problem, a two-year field experiment was conducted to investigate the effects of different irrigation and nitrogen applications on soil water storage, water consumption, jujube yield, and fruit quality, as well as water and nitrogen use efficiency under STI, and to determine the optimal irrigation and nitrogen application solution by the entropy weight-based TOPSIS method.

**Results:**

The results showed that irrigation very significantly (*p* < 0.01) affected average soil water storage under STI over the entire growth period. STI increased jujube yield significantly (*p* < 0.05) by 18.1% in 2021 and 15.5% in 2022 compared with surface drip irrigation at the same irrigation and nitrogen amount, respectively. Both irrigation and nitrogen very significantly (*p* < 0.01) affected crop evapotranspiration (ETa), jujube yield, and nitrogen partial factor productivity (NPFP) in both years. The jujube yield increased linearly with irrigation amount, while showing a unimodal trend with nitrogen application. A significant linear relationship (*p* < 0.01) between ETa and jujube yield was observed in both years. Nitrogen application significantly affected water use efficiency in both years. In addition, STI increased fruit vitamin C content and sugar-acid ratio to some extent compared with surface drip irrigation. However, the overall effect of irrigation water and nitrogen on each quality index was not significant under STI. Under the same irrigation and nitrogen conditions, STI increased average net income by 51.3% compared with surface drip irrigation in both years. Through a comprehensive analysis of the binary quadratic regression equation and entropy weight-based TOPSIS method, the optimal irrigation and nitrogen amounts were 3600 m^3^ ha^-1^ and 370 kg ha^-1^, respectively.

**Discussion:**

The research will provide technical support for the optimal agricultural water and nitrogen management of jujube in arid areas.

## 1 Introduction

Freshwater scarcity has directly affected food production and led to food safety problems worldwide (Falkenmark, 2013; Rehman et al., 2022; Rockström et al., 2009). Total global food supply is projected to increase by 30%–62% by 2050 compared with 2010 (Van Dijk et al., 2021). Improvement of agricultural water productivity is one of the critical options to address food security (Brauman et al., 2013; Kang et al., 2017; Mozumdar, 2012; Rehman et al., 2022). Innovations in water-saving irrigation technologies are key to improving agricultural water productivity.

Nowadays, drip irrigation is the most commonly-used water-saving irrigation technology and has been widely reported as increasing crop yield, enhancing water productivity, and improving soil environment (Cao et al., 2022; Li et al., 2021; Zong et al., 2023). Surface drip irrigation saves water compared with conventional flood, sprinkler and furrow irrigation, but its associated surface ponding also leads to considerable water evaporation. STI is a type of indirect subsurface drip irrigation that is effective in reducing surface wetting and increasing soil water storage in the lower soil layers by rapidly directing irrigation water through fine sand to the plant rhizosphere (Bai et al., 2022; Meshkat et al., 1998, 2000; Sun et al., 2016). Reducing surface evaporation and increasing soil water storage are the key reasons why STI enhances jujube yield in arid regions (Bai et al., 2022; Meshkat et al., 1999). STI cultivation, therefore, has a bright future for use in forest and fruit production in arid areas.

Water and nitrogen are the two main limiting factors for crop growth and productivity (Plett et al., 2020; Sinclair and Rufty, 2012). Soil moisture deficit can decrease the flow and mineralization of nitrogen in the soil, as well as its transport in the plant, thus reducing plant nitrogen use efficiency (He and Dijkstra, 2014; Marino et al., 2007). Nowadays, it is extremely common for farmers to apply large amounts of additional nitrogen fertilizer in order to increase crop production (Kang et al., 2017; Lassaletta et al., 2023). It is worthwhile, therefore, to determine a reasonable amount of irrigation and nitrogen based on the long-term cultivation experience of local farmers.

Ridge and furrow mulching can effectively improve net productivity in semi-arid agriculture areas and has been widely used (Gan et al., 2013; Gao et al., 2019). However, in arid areas, especially in arid desert areas, there are fewer studies on nitrogen regulation by ridge and furrow mulching combined with STI. There may be a room for further improvement of yield and economic benefits, when compared with traditional surface drip irrigation or furrow drip irrigation.

The jujube tree has been cultivated in China for over 4,000 years. The jujube fruit is rich in nutrients, and analyses by phytochemical researchers has shown the fruit to contain a variety of components such as proteins, vitamins, sugars, organic acids and minerals. These play a significant role in the prevention of oxidative stress, especially the high content of vitamin C, which is known as a natural “vitamin pill” (Shi et al., 2022; Wang et al., 2012; Wojdyło et al., 2016). Jujube is valued for its attractive taste and health benefits such as reducing triglycerides, low-density lipoprotein, and cholesterol and is widely processed into products like cakes and yogurt, as well as used in traditional medicine and animal feed (Hoseinifar et al., 2018; Song et al., 2019) (Cellat et al., 2022; Liu et al., 2019; Zhang et al., 2018; Zhu et al., 2024). In addition, the jujube tree is planted as a major ecological tree species in arid zones due to its drought tolerance and low soil requirements (Liu et al., 2020). The jujube tree has, therefore, a significant role in industrial and agricultural production, as well as in economic development and ecological protection.

In arid regions, jujube trees are commonly cultivated under surface drip irrigation, which often results in high surface evaporation and low water and fertilizer use efficiency (Wang et al., 2021). Appropriate irrigation and fertilization amounts are important factors for increasing jujube yield and improving fruit quality (Dai et al., 2019). The application of ridge and furrow mulching combined with STI, therefore, has a valuable role for the cultivation of jujube tree in arid areas. Previous studies on STI have mainly focused on single factors, such as the effects of irrigation alone on yield and water use efficiency (Bai et al., 2022; Sun et al., 2016), while few have systematically investigated the coupled effects of irrigation and nitrogen management on soil water storage, water consumption, fruit quality, and resource use efficiency. Moreover, the optimal combination of irrigation and nitrogen application under this system remains unclear, especially in arid regions. The main objectives of this study were to: (1) investigate the effects of different irrigation and nitrogen application on soil water storage, water consumption, jujube yield and quality, as well as water and nitrogen use efficiency under STI, and (2) determine the optimal irrigation and nitrogen application solution by entropy weight-based TOPSIS. This study aims to provide a scientific basis for water−saving irrigation and nitrogen management under STI in arid regions, as well as a practical evaluation method for multi−objective optimization.

## 2 Materials and methods

### 2.1 Study site

**Figure 1:**
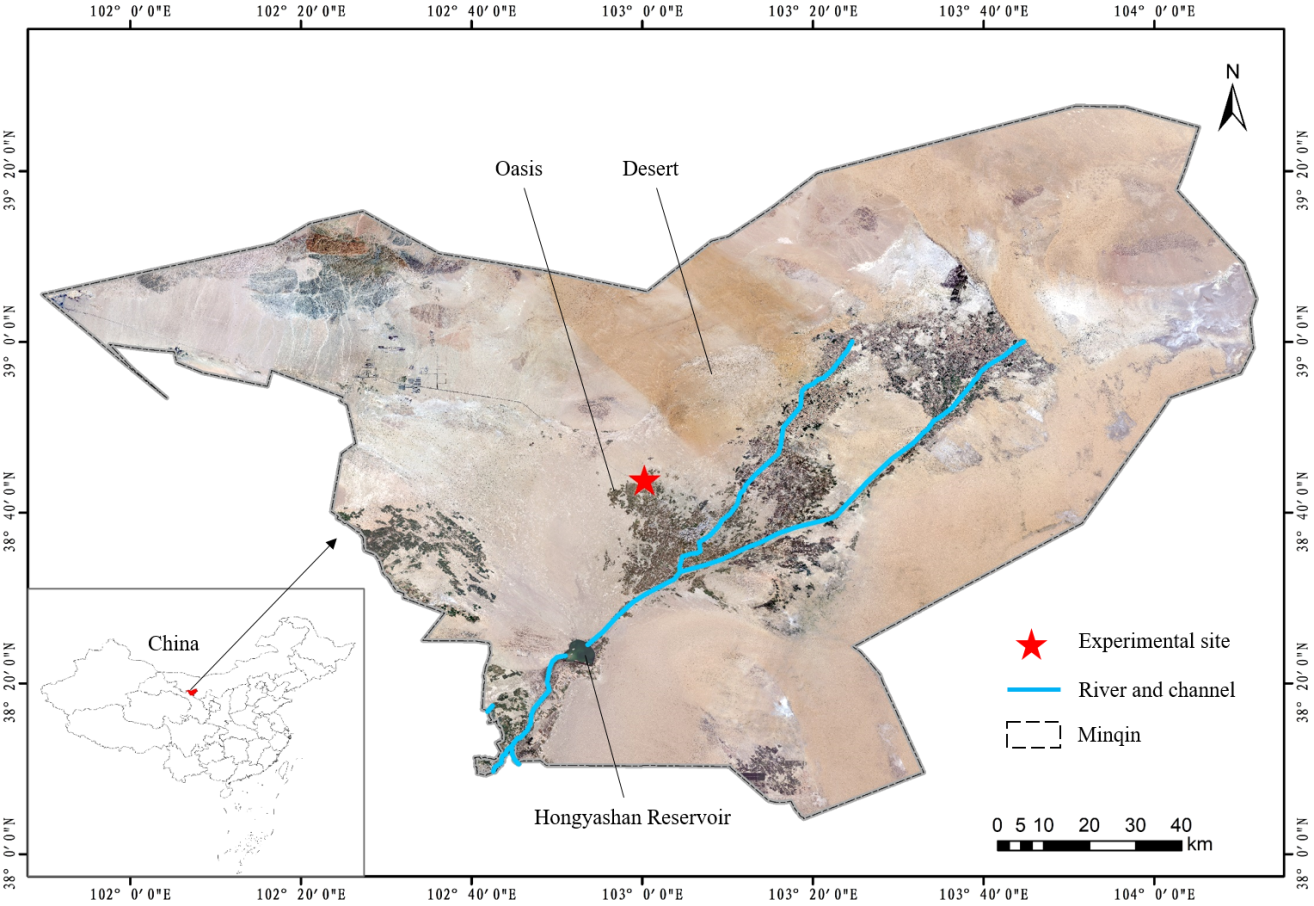
Location of the research area.

The field experiment was set up at Qinfeng Forestry Experimental Station (38^°^43′54″N,103^°^01′05″E) in Minqin County, Gansu Province, China (**Figure 1**). The study site is located in the hinterland of Badain Jaran and Tengger Deserts and has a continental desert-dry with low rainfall and high evaporation. The annual rainfall and evaporation are 110 mm and 2644 mm, respectively. The average annual temperature is 7.8 °C. The depth of groundwater is below 40 m. Soil particle size classification and physical properties are shown in **Table 1**.

**Table 1:** Soil physical parameters at different depths from 0 to 100 cm.

Soil depth (cm)	Soil particle size composition(mm)			Bulk density (g cm^−3^)	Field capacity (mass water content, %)
	< 0.002	0.002–0.05	0.05–2		
0–20	14.87	48.77	36.36	1.43 ± 0.13	20.0
20–40	16.39	51.44	32.17	1.56 ± 0.07	
40–60	16.98	43.27	39.74	1.65 ± 0.06	
60–100	17.22	69.75	13.03	1.51 ± 0.15	16.17

### 2.2 Experimental design

**Figure 2:**
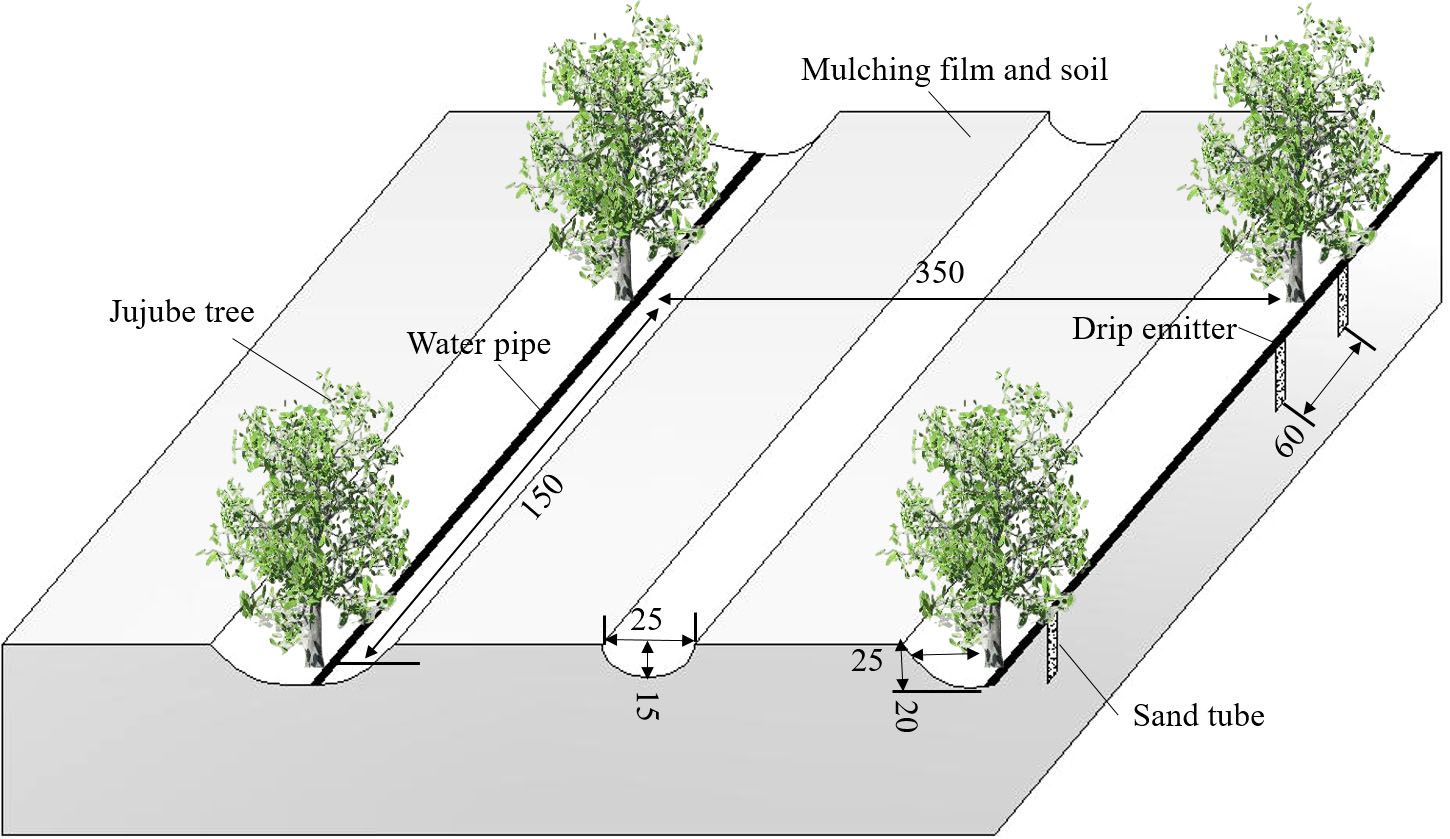
Arrangement of sand tube irrigation.

Eight-year-old jujube trees (*Ziziphus jujuba Mill*. Jun-jujube) were used for the field experiments during the jujube growth period from May to October in 2021 and 2022. Trees spacing was 150 cm and rows was 350 cm apart. A split-plot experiment (**Figure 2**) was performed with three irrigation levels (low water, W_1_, 300 m^3^ ha^–1^; medium water, W_2_, 375 m^3^ ha^–1^; high water, W_3_, 450 m^3^ ha^–1^) as the main plots and four nitrogen levels (F_1_, 0; low nitrogen, F_2_, 286 kg ha^–1^; medium nitrogen, F_3_, 381 kg ha^–1^; high nitrogen, F_4_, 476 kg ha^–1^) as the subplots for STI treatments. Traditional surface drip irrigation (irrigation at 450 m^3^ ha^–1^ and nitrogen at 476 kg ha^–1^) was used as a control (CK). In order to effectively maintain soil moisture and reduce the growth of weeds, the ridge was covered with plastic film, then a 2–3 cm soil layer placed on top of the film, and the jujube trees were planted in the furrows. A water supply pipeline with drip emitters (flow rate: 4L h^–1^) was laid in the furrow. A cylindrical sand tube (diameter: 10 cm and depth: 20 cm) was installed directly under the drip emitter. Sand tubes filled with fine sand (particle diameter: 2-3mm) were set up on both sides of the jujube tree. A water meter (accurate to 0.0001 m^3^) was used to control each irrigation level. Irrigation water was from a local supply well and the water quality met irrigation requirements.

Field irrigation and nitrogen strategies in 2021 and 2022 are shown in **Table 2**. Irrigation was carried out eight times during the entire growth periods in 2021 and 2022. Irrigation dates were the same for all treatments. Nitrogen was carried out three times during the entire growth periods, at a rate of 30%, 30%, and 40% of the total N at the beginning of flowering, at the end of flowering and at the beginning of fruit swelling, respectively.

**Table 2:** Field irrigation and nitrogen strategies in 2021 and 2022.

Year	Item	Treatment	1st irrigation	2nd irrigation	3rd irrigation	4th irrigation	5th irrigation	6th irrigation	7th irrigation	8th irrigation
2021	Irrigation date		5.11	6.1	6.21	7.6	7.21	8.6	8.21	9.6
	Irrigation amount (m^3^ ha^-1^)	W1	300	300	300	300	300	300	300	300
		W2	375	375	375	375	375	375	375	375
		W3	450	450	450	450	450	450	450	450
		CK	450	450	450	450	450	450	450	450
	Nitrogen ratio (total N%)			30%	30%	40%				
2022	Irrigation date		5.7	5.28	6.18	7.4	7.2	8.6	8.19	9.1
	Irrigation amount (m^3^ ha^-1^)	W1	300	300	300	300	300	300	300	300
		W2	375	375	375	375	375	375	375	375
		W3	450	450	450	450	450	450	450	450
		CK	450	450	450	450	450	450	450	450
	Nitrogen ratio (total N%)			30%	30%	40%				

### 2.3 Monitoring indicators and calculating parameters

#### 2.3.1 Meteorological conditions

**Figure 3:**
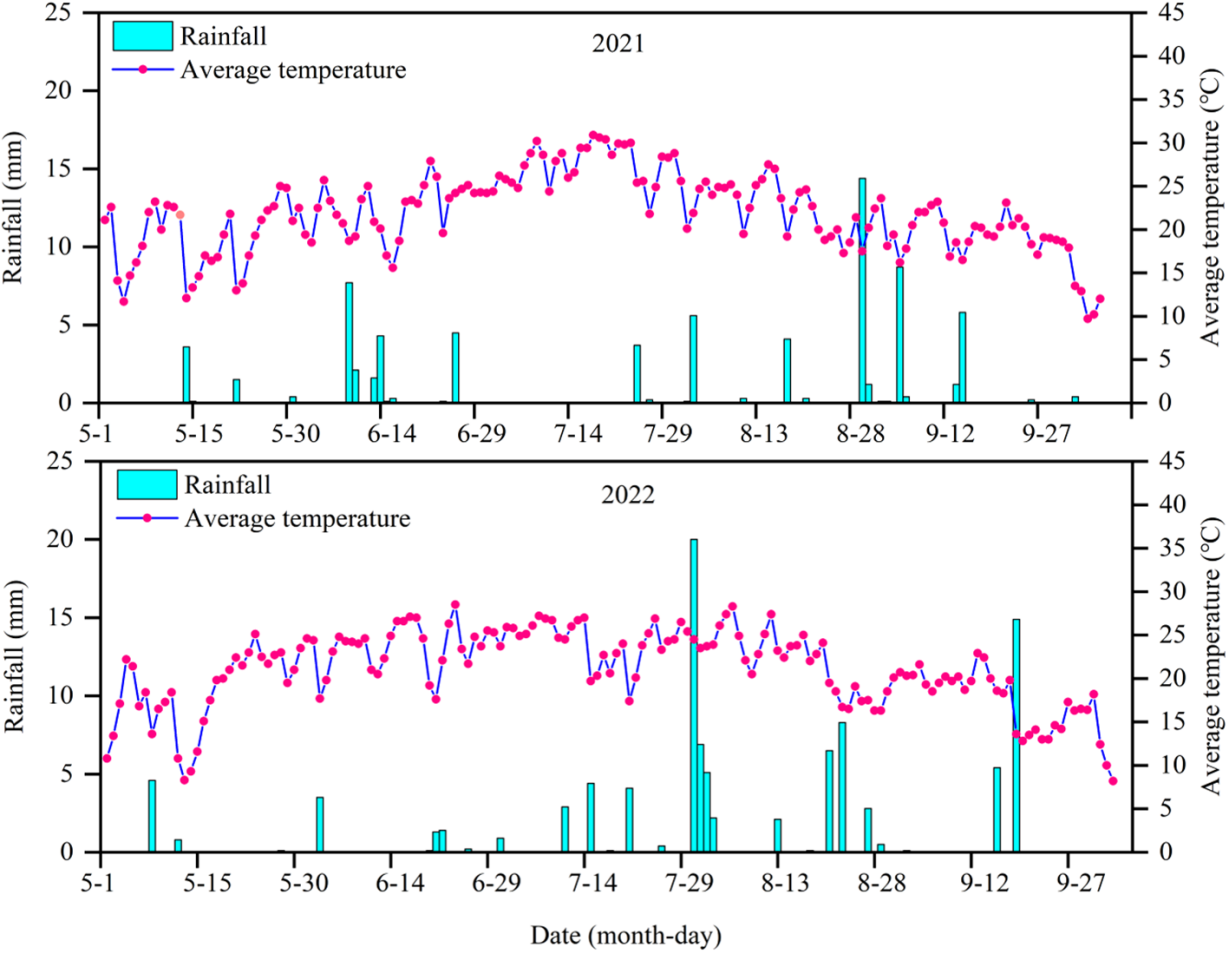
Daily rainfall and average temperature during the growth period of jujube trees in 2021 and 2022.

A meteorological monitoring station (TRM-ZS2, Sunshine Meteorology Co., LTD., China) was installed at the experimental site. It can monitor rainfall, air temperature, solar radiation, air pressure, relative air humidity, and wind speed at 30-min intervals. Rainfall was 82.5 mm (effective rainfall, 50.2 mm) and 99.7 mm (effective rainfall, 67.1 mm) in 2021 and 2022, respectively, during the whole growth periods. The average air temperature during the growth period was 21.8 and 21.0 °C in 2021 and 2022, respectively (**Figure 3**).

#### 2.3.2 Soil water content

The main water-absorbing roots (diameter< 2 mm) of jujube trees, which function as the dominant organs for water and nutrient uptake, are primarily distributed in the 0–60 cm soil layer (Montagnoli et al., 2012; Wang et al., 2015). Therefore, soil samples were collected from the 0–100 cm soil layer using an iron soil auger (inner diameter: 4.0 cm). Soil water content (gravimetric, %) was determined at 10 cm increments to a depth of 100 cm using the oven–drying method before and after irrigation, and after harvesting.

Soil water storage (SWS, mm) was calculated using Equation 1 (Liu et al., 2018).

(1) $$SWS=\sum_{i=1}^{10}\left(h_{i}\times a_{i}\times \theta_{i}\right)\times 10$$

where *h* is the soil layer thickness (10 cm), *a* is the soil bulk density (g cm^–3^), and *θ* is the soil water content (gravimetric, %), *i* is the soil layer, and 10 is the unit conversion coefficient.

#### 2.3.3 Crop evapotranspiration (ET_a_)

Crop evapotranspiration (ET_a_, mm) was determined through the water balanced equation (Liang et al., 2019):

(2) $$ET_{a}=I+P+\Delta W-D-R$$

where *I*, *P*, and Δ*W* are the irrigation amount (mm), the effective rainfall, and the depletion of SWS in the 0–100 cm soil layer. *D* and *R* are the deep seepage and the water runoff, respectively. Both are negligible because of the use of STI technology and weak rainfall intensity, which is less prone to deep seepage and surface runoff.

#### 2.3.4 Jujube yield, fruit quality

Jujube yield (*Y*, kg ha^–1^) was determined by calculating the average of the total weight of jujube fruit per tree for each treatment. Jujube fruits of each treatment were harvested on October 7–8, 2021, and October 4–5, 2022. Each quality index was determined by selecting 6–8 jujube fruits from each tree in 4 directions: east, west, south, and north. Vitamin C (Vc) was measured by titration with 2,6-dichlorophenol indophenol sodium. Soluble total sugar content was determined using the anthrone colorimetric method. Soluble protein was determined by the Bradford method with Coomassie brilliant blue G250. Total organic acid content was measured by acid–base titration method. Soluble solid content was measured using a PAL-1 refractometer (0–53%, Atago, Tokyo, Japan).

#### 2.3.5 Water use efficiency, irrigation water use efficiency, and nitrogen partial factor productivity

The WUE (kg m^−3^) and IWUE (kg m^−3^) were determined through the following two formulas (Bai et al., 2024):

(3) $$WUE=\frac{Y}{ET_{a}}$$

(4) $$IWUE=\frac{Y}{I}$$

The NPFP, (kg kg^−1^) was determined through the following formula (Ma et al., 2022):

(5) $$NPFP=\frac{Y}{N}$$

where Y, ET_a_, I, and N are the jujube yield (kg ha^–1^), crop evapotranspiration (mm), irrigation amount (mm), and the total nitrogen amount (kg ha^–1^) for each treatment.

### 2.4 Economic benefit analysis

The economic benefit analysis was calculated as follows:

(6) $$E_{net}=T_{output}-T_{input}$$

Where *E_net_* is the net income (¥ ha^–1^), *T_output_* is the total output (mainly from jujube fruits, ¥ ha^–1^), *T_input_* is the total input (mainly including water fees, fertilizers, drip irrigation systems, and labor expenses, ¥ ha^–1^). Specifically, the market price of jujube was 6.67 ¥ kg^–1^, irrigation water cost was 0.342 ¥ m^–3^, and urea was price at 1.90 ¥ kg^–1^. Labor expenditure was estimated according to the local wage standard, with male laborers paid at 150 ¥ day^−1^ for an 8-working-hour daily shift.

### 2.5 Determination of optimal irrigation and nitrogen fertilization strategy based on entropy weight and TOPSIS methods

The TOPSIS method was used to find a solution in the feasible solution set by defining the positive ideal solution and the negative ideal solution of the decision problem; then finally, choosing the ideal solution that is most positive and furthest from the negative ideal solution. The analytical steps are as follows (Hwang and K, 1981; Kim, 2016; Shih et al., 2007):

(1) Build the comprehensive evaluation indicator system and determine the data matrix X. The evaluation objects are the treatments and the evaluation indicators are the Jujube yield, WUE, IWUE, NPFP, Vc, Total organic acid, Total input, Total output, and Net income. Among them, the positive indicators are Jujube yield, WUE, IWUE, NPFP, Vc, Total output, and Net income, while the negative indicators are Total organic acid and Total input.

(7) $$X=\left[\begin{array}{ccccc} x_{11} & \ldots & x_{1j} & \ldots & x_{1n}\\ \ldots & \ldots & \ldots & \ldots & \ldots \\ x_{i1} & \ldots & x_{ij} & \ldots & x_{in}\\ \vdots & \vdots & \vdots & \vdots & \vdots \\ x_{m1} & \ldots & x_{mj} & \ldots & x_{mn} \end{array}\right]$$

Where *x_ij_* denotes the jth evaluation indicator for the ith treatment of original data, and m (treatments), n (evaluation indicators) are 10, and 9, respectively.

(2) Standardize evaluation indicators and construct the normalized matrix P. The formulae are below:

(8) $$p_{ij}=\frac{x_{ij}}{\sqrt{\sum_{i=1}^{m}{\left(x_{ij}\right)}^{2}}}$$

(9) $$P={\left(p_{ij}\right)}_{m\times n}=\left[\begin{array}{ccccc} p_{11} & \ldots & p_{1j} & \ldots & p_{1n}\\ \ldots & \ldots & \ldots & \ldots & \ldots \\ p_{i1} & \ldots & p_{ij} & \ldots & p_{in}\\ \vdots & \vdots & \vdots & \vdots & \vdots \\ p_{m1} & \ldots & p_{mj} & \ldots & p_{mn} \end{array}\right]$$

Where *p_ij_* is the contribution of jth evaluation indicator for the ith treatment.

(4) Calculate the entropy value of the jth indicator.

(10) $$e_{j}=-k\sum_{i=1}^{m}p_{ij}\left(\ln p_{ij}\right)$$

where *e _j_* is the entropy value of the jth metric across all treatments, and k = 1/ln(n).

(5) Calculate the difference coefficient *d_j_* for the jth indicator.

(11) $$d_{j}=1-e_{j}$$

(6) Calculate the weight of each evaluation indicator.

(12) $$w_{j}=\frac{d_{j}}{\sum_{j=1}^{n}d_{j}}$$

(7) Construct a weight-based normalized matrix.

(13) $$Z={\left(z_{ij}^{\prime }\right)}_{m\times n}=P\times w_{j}$$

(8) Determine the positive ideal solution vector *Z*^+^and the negative ideal solution vector *Z*^−^.

(14) $$\begin{array}{l} Z^{+}=\left(Z_{1}^{+},Z_{2}^{+},\ldots Z_{n}^{+}\right)\\ =\left(\max\left\{z_{11}^{\prime },z_{21}^{\prime },\ldots Z_{m1}^{\prime }\right\},\max\left\{z_{12}^{\prime },z_{22}^{\prime },\ldots Z_{m2}^{\prime }\right\},\ldots ,\max\left\{z_{1n}^{\prime },z_{2n}^{\prime },\ldots Z_{mn}^{\prime }\right\}\right) \end{array}$$

(15) $$\begin{array}{l} Z^{-}=\left(Z_{1}^{-},Z_{2}^{-},\ldots Z_{n}^{-}\right)\\ =\left(\min\left\{z_{11}^{\prime },z_{21}^{\prime },\ldots Z_{m1}^{\prime }\right\},\min\left\{z_{12}^{\prime },z_{22}^{\prime },\ldots Z_{m2}^{\prime }\right\},\ldots ,\min\left\{z_{1n}^{\prime },z_{2n}^{\prime },\ldots Z_{mn}^{\prime }\right\}\right) \end{array}$$

(9) Calculate the euclidean distances $D_{i}^{+}$ and $D_{i}^{-}$ between each evaluation indicator, and the relative closeness to ideal solution *C_i_*.

(16) $$D_{i}^{+}=\sqrt{\sum_{j=1}^{n}{\left(Z_{j}^{+}-z_{ij}^{\prime }\right)}^{2}}$$

(17) $$D_{i}^{-}=\sqrt{\sum_{j=1}^{n}{\left(Z_{j}^{-}-z_{ij}^{\prime }\right)}^{2}}$$

(18) $$C_{i}=\frac{D_{i}^{-}}{D_{i}^{+}+D_{i}^{-}}$$

(10) Rank the preference order

In this step, the *C_i_* values of each treatment are ranked from largest to smallest, and the closer to 1 is the optimal option.

### 2.6 Statistical analysis

Data were tested by analysis of variance (ANOVA) using SPSS 22 statistical software (SPSS Inc., Chicago, USA). Prior to the analysis, the assumptions of homogeneity of variances and normality were checked and met (*p* > 0.05). Comparison of means was carried out using the LSD method at the 5% level of signification. The 2021 and 2022 experiments were independent field trials; data from each year were analyzed separately, with no combined analysis across years. Origin 2024 (Origin Lab, MA, USA) was used for mapping and the fitting of linear equations. The TOPSIS method was used to make and evaluate the multi-objective decision.

## 3 Results

### 3.1 Average SWS of entire growth period

**Figure 4:**
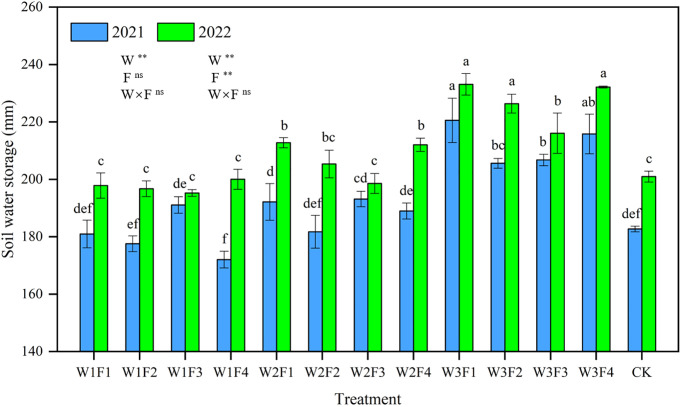
Changes in average soil water storage over the entire growth periods for each treatment in 2021 and 2022. Different lowercase letters indicate significant differences at *p*< 0.05. Bars indicate the standard error.

Irrigation can effectively replenish soil moisture and affect average SWS (Equation 1) of entire growth period very significantly (*p*< 0.01, **Figure 4**). As shown in **Figure 4**, average SWS increased with the increase of irrigation water in both years under STI. In 2021, each of the W3 treatments was increased significantly (*p*< 0.05) by 8.2%–25.5% and 7.0%–14.8% compared with each of the W1 and W2 treatments, respectively. In 2022, the former was increased significantly (*p*< 0.05) by 10.7%–17.8% and 8.8%–10.2% compared with the latter two, respectively. STI (W3 treatments) increased significantly (*p*< 0.05) 12.5%–20.7% in 2021 and 7.5%–16.0% in 2022), compared with surface drip irrigation under the same irrigation amount (CK). STI increased significantly (*p*< 0.05) by 18.1% in 2021 and 15.5% in 2022 compared with surface drip irrigation at the same irrigation and nitrogen amount, respectively. In addition, the average SWS of W3 treatment tended to decrease and then slowly increase with increasing nitrogen application. The W3F1 treatment showed the largest in both years and the smallest values were in the W3F2 treatment (2021) and W3F3 treatment (2022).

### 3.2 ET_a_, jujube yield, WUE, IWUE, and NPFP

**Figure 5:**
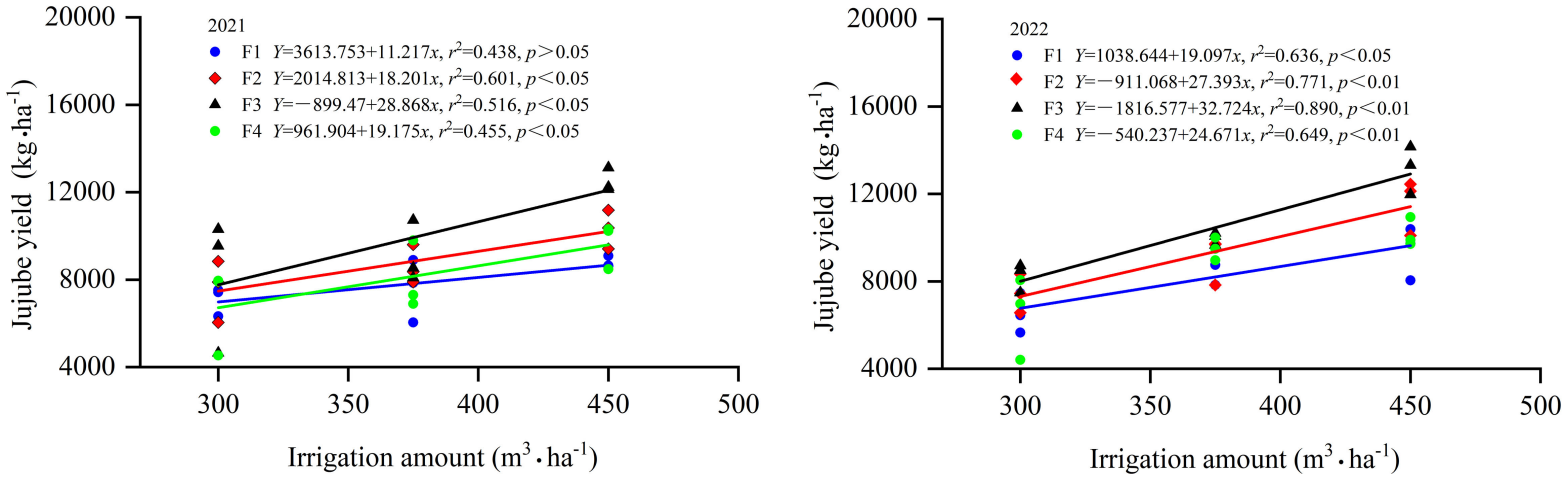
Relationship between irrigation amount and jujube yield in 2021 and 2022.

**Figure 6:**
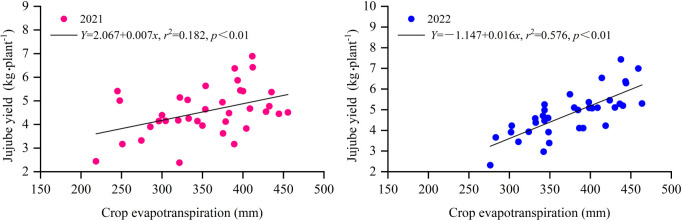
Relationship between crop evapotranspiration and jujube yield in 2021 and 2022.

ANOVA for ET_a_ (Equation 2), jujube yield, WUE (Equation 3), IWUE (Equation 4), and NPFP (Equation 5) of each treatment in 2021 and 2022 are shown in **Table 3**. Both irrigation and nitrogen affected very significantly (*p*< 0.01) ET_a_ in 2021 and 2022, and ET_a_ tended to increase with increasing irrigation in both years. The treatments with the largest ET_a_ were CK in 2021 and W3F3 in 2022, while the minimum values were in W1F3 and W1F4, respectively. Moreover, irrigation and nitrogen affected jujube yield very significantly (*p*< 0.01) in 2021 and 2022. Under STI, jujube yield tended to increase in a linear with increasing irrigation amount (**Table 3**; **Figure 5**). In addition, jujube yield tended to increase and then decrease with increasing nitrogen under STI in both years (**Table 3**). Maximum jujube yield (12495.23 kg ha^–1^ in 2021 and 13142.84 kg ha^–1^ in 2022) were for the treatment with maximum irrigation and nitrogen of F3 (W3F3) in both years. Furthermore, W3F3 significantly (*p*< 0.05) increased jujube yield 74.5% in 2021 and 71.6% in 2022 compared with CK. In addition, W3F3 significantly (*p*< 0.05) increased jujube yield in both years compared with each of the W1 and W2 treatments. W2F3 (20% water and 25% nitrogen reduction) increased yield by 27.4% (2021, *p*>0.05) and 30.3% (2022, *p*< 0.05) compared with CK. Moreover, there was a significant linear relationship (*p*< 0.01) between ET_a_ and jujube yield in both years (**Figure 6**).

**Table 3:** Analysis of variance for ETa, jujube yield, WUE, IWUE, and NPFP of each treatment in 2021 and 2022.

Treatment	Effective rainfall (≥5 mm)		ET_a_ (mm)		Yield (kg ha^–1^)		WUE (kg m^−3^)		IWUE (kg m^−3^)		NPFP (kg kg^−1^)	
	2021	2022	2021	2022	2021	2022	2021	2022	2021	2022	2021	2022
W1F1	50.2	67.1	303.41 g	346.67 fg	7085.71 cd	6514.28 g	2.35 bcd	1.88 cd	2.96 ab	2.72 cde	–	–
W1F2	50.2	67.1	300.32 g	315.31 hi	7580.94 bcd	7447.61 fg	2.52 abc	2.36 bcd	3.16 a	3.1 abcd	26.55 bcd	26.07 c
W1F3	50.2	67.1	237.16 h	333.16 gh	8171.42 bcd	8247.61 defg	3.39 a	2.47 ab	3.40 a	3.43 ab	21.43 def	21.63 cd
W1F4	50.2	67.1	315.44 fg	287.52 i	6799.99 d	6476.18 g	2.16 cd	2.24 bcd	2.83 ab	2.7 cde	14.27 f	13.6 f
W2F1	50.2	67.1	392.18 bcd	373.03 ef	7599.99 bcd	8685.71 cdef	1.93 cd	2.34 bcd	2.53 ab	2.9 bcd	–	–
W2F2	50.2	67.1	325.25 efg	390.02 de	8609.52 bcd	9085.71 cdef	2.66 abc	2.33 bcd	2.87 ab	3.03 abcd	30.16 abc	31.77 b
W2F3	50.2	67.1	356.61 def	412.27 bcd	9123.80 bcd	9980.94 bcd	2.56 abc	2.42 abc	3.04 a	3.33 abc	23.93 cde	26.20 c
W2F4	50.2	67.1	367.27 de	342.86 gh	7999.99 bcd	9485.70 cde	2.23 bcd	2.77 ab	2.66 ab	3.16 abcd	16.79 ef	19.91 de
W3F1	50.2	67.1	438.86 ab	424.40 abc	8780.94 bcd	9390.47 cde	2.00 cd	2.21 bcd	2.44 ab	2.61 de	–	–
W3F2	50.2	67.1	388.30 cd	440.49 ab	10323.80 ab	11561.89 ab	2.65 abc	2.64 ab	2.87 ab	3.21 abcd	36.11 a	40.45 a
W3F3	50.2	67.1	404.56 bcd	447.02 a	12495.23 a	13142.84 a	3.09 ab	2.94 a	3.47 a	3.65 a	32.8 ab	34.51 b
W3F4	50.2	67.1	426.63 abc	408.00 cd	9676.18 bc	10171.42 bc	2.28 bcd	2.51 ab	2.69 ab	2.83 bcd	20.31 def	21.37 cd
CK	50.2	67.1	457.51 a	427.82 abc	7161.90 cd	7657.14 efg	1.57 d	1.79 d	1.99 b	2.12 e	15.04 f	16.06 ef
W	/	/	**	**	**	**	ns	*	ns	ns	**	**
F	/	/	**	**	**	**	**	*	ns	**	**	**
W×F	/	/	ns	ns	ns	ns	ns	ns	ns	ns	ns	ns

"/", not tested; *, p < 0.05; **, p < 0.01.

Considering WUE, nitrogen increased it very significantly (*p*< 0.01) in 2021, and both irrigation and nitrogen increased it significantly (*p*< 0.05) in 2022 under STI (**Table 3**). The largest WUE values were from the W1F3 (2021) and W3F3 (2022) treatments, respectively, while the smallest were both from CK. The W1F3 was not significantly different from the W3F3 treatment in both years, but both were significantly (*p*< 0.05) different from CK. WUE increased by 45.2% (2021, *p*>0.05) and 40.22% (2022, *p*< 0.05) under STI compared with CK under the same irrigation and nitrogen, respectively. STI under F3 conditions were significantly (*p*< 0.01) increased from CK by 63.1%–115.9% in 2021 and 35.2%–64.3% in 2022.

For IWUE, in both years, the increase in irrigation did not have significant impact on IWUE. The treatment with the highest IWUE was W3F3, and it was significantly (*p*< 0.05) higher than CK by 74.4% (2021) and 72.2% (2022), respectively. IWUE increased by 35.2% (2021, *p*>0.05) and 33.5% (2022, *p*< 0.05) under STI compared with CK under the same irrigation and nitrogen, respectively.

For NPFP, irrigation and nitrogen affected the indicator very significantly (*p*< 0.01) in 2021 and 2022. Under STI, NPFP tended to decrease with increasing nitrogen over the two years. Under the same irrigation and nitrogen application conditions, STI increased by 35.0% (2021, *p*>0.05) and 33.1% (2022, *p*< 0.05), respectively, compared with CK. The treatment with the largest NPFP was W3F2, followed by W3F3 and the lowest was from W1F4 in both years.

### 3.3 Jujube fruit quality

Comparisons of jujube fruit quality for each treatment in 2021 and 2022 are shown in **Table 4**. A comprehensive analysis shows that fruit quality parameters showed limited and inconsistent treatment effects across years. Statistical results show that the Vc of W3F3 was significantly higher (*p*< 0.05) than that of CK in both 2021 and 2022. Irrigation and nitrogen did not significantly affect the treatments under STI. In addition, W1F1 was significantly (*p*< 0.05) different from CK, but not from W1F3. For soluble total sugar, the treatments as a whole did not differ significantly, and the minimum values were W3F3 (2021) and W2F2 (2022). In 2021, the minimum value of soluble protein is W3F1, which is significantly (*p*< 0.05) different from W1F3. In 2022, the minimum value was W3F4, which was significantly (*p*< 0.05) different from W2F2 and W2F2. The maximum value of total organic acid content was from CK in both years and showed significant difference from W1F3, W2F3 (2021) and W2F2 (2022). In addition, in both years, the W3F3 treatment increased soluble solid by 30.6% and 46.8%, respectively, compared with CK. In 2021, the difference between the treatments as a whole was not significant. However, STI led to a significant (*p*< 0.05) increase by 27.2%–56.0% in 2022 compared with CK (except for W1F2, W1F4, and W2F1). Moreover, for sugar-acid ratio, the largest treatments in both years were W1F3 and W3F2, respectively, and the differences were significant (*p*< 0.05) compared with CK. However, STI with different irrigation and nitrogen had no significant effect on sugar-acid ratio as a whole.

**Table 4:** Comparisons of jujube fruit quality for each treatment in 2021 and 2022.

Treatment	2021						2022					
	Vc (mg 100g^-1^)	Soluble total sugar (%)	Soluble protein (mg g^-1^)	Total organic Acid (%)	Soluble solid (%)	Sugar–acid ratio	Vc (mg 100g^-1^)	Soluble total sugar (%)	Soluble protein (mg g^-1^)	Total organic Acid (%)	Soluble solid (%)	Sugar–acid ratio
W1F1	42.19 a	0.58 ab	0.2 ab	0.40 ab	20.2 ab	49.80 b	63.21 a	0.58 a	0.15 bc	0.64 abc	31.91 abc	51.07 ab
W1F2	32.63 abc	0.61 ab	0.18 ab	0.44 ab	21.04 ab	47.91 b	51.08 ab	0.54 ab	0.14 c	0.69 abc	26.31 cd	40.17 bc
W1F3	33.31 abc	0.66 ab	0.25 a	0.36 b	27.45 ab	76.47 a	54.99 ab	0.49 ab	0.17 abc	0.77 ab	32.51 abc	42.99 abc
W1F4	30.75 abc	0.65 ab	0.21 ab	0.46 ab	23.98 ab	56.58 ab	52.34 ab	0.5 ab	0.16 abc	0.63 abc	26.08 cd	42.86 abc
W2F1	36.77 ab	0.68 ab	0.2 ab	0.48 ab	22.41 ab	49.30 b	52.73 ab	0.5 ab	0.17 abc	0.69 abc	29.68 bcd	49.33 ab
W2F2	30.78 abc	0.68 ab	0.2 ab	0.41 ab	23.11 ab	56.77 ab	43.9 ab	0.4 b	0.2 a	0.53 c	30.08 bc	57.39 ab
W2F3	31.17 abc	0.64 ab	0.19 ab	0.37 b	18.56 b	49.23 b	58.51 a	0.56 a	0.2 ab	0.71 abc	34.08 ab	48.67 ab
W2F4	22.69 c	0.71 a	0.19 ab	0.39 ab	24.35 ab	62.59 ab	56.44 ab	0.47 ab	0.16 abc	0.67 abc	30.32 bc	45.43 abc
W3F1	32.2 abc	0.62 ab	0.16 b	0.48 ab	21.57 ab	49.65 b	43.76 ab	0.56 a	0.16 abc	0.6 bc	31.22 abc	52.6 ab
W3F2	38.85 ab	0.64 ab	0.21 ab	0.41 ab	21.08 ab	52.77 ab	56.64 ab	0.58 a	0.16 abc	0.59 bc	36.89 a	62.15 a
W3F3	43.8 a	0.56 b	0.19 ab	0.44 ab	28.18 a	64.18 ab	61.1 a	0.49 ab	0.16 abc	0.72 abc	34.72 ab	49.33 ab
W3F4	31.14 abc	0.63 ab	0.18 ab	0.44 ab	23.24 ab	52.97 ab	49.44 ab	0.52 ab	0.14 c	0.65 abc	30.85 abc	47.17 abc
CK	26.56 bc	0.62 ab	0.22 ab	0.56 a	21.57 ab	38.97 b	32.79 b	0.5 ab	0.16 abc	0.85 a	23.65 d	28.22 c

Different letters within a column indicate p < 0.05.

### 3.4 Benefit analysis

**Figure 7:**
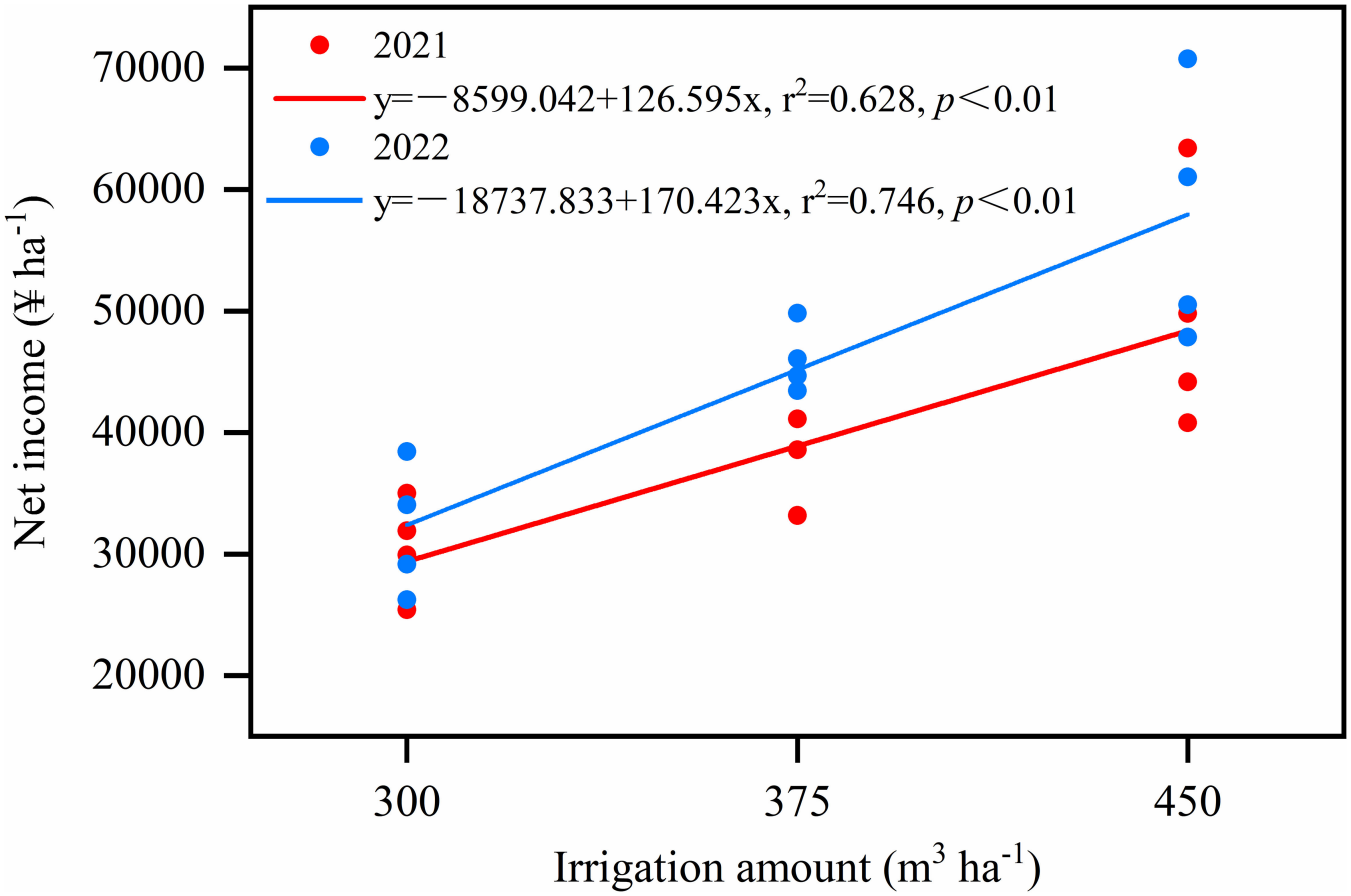
Relationship between irrigation amount and net income in 2021 and 2022.

Technological innovation is an important stimulus to increased agricultural productivity and poverty reduction (Alene and Coulibaly, 2009; Dhrifi, 2014). Total inputs (including consumables, water coasts, labor costs, etc.) are higher for STI compared with surface drip irrigation (Equation 6; **Table 5**). The yield of STI is higher compared with surface drip irrigation at the same or a reduced irrigation amount (**Table 3**). Statistical results showed that STI still increased total output by 6.1%–67.7% (2021) and 13.7%–71.9% (2022) with 20% reduction in irrigation water or under the same conditions. Similarly, STI increased net income by 14.7%–119.3% (2021) and 29.1%–110.3% (2022) when irrigation was reduced by 20% or under the same conditions. Under the same irrigation and nitrogen conditions, STI increased average net income by 51.3% compared with surface drip irrigation. In addition, net income increased with increasing irrigation amount under STI (**Figure 7**). In conclusion, under the same irrigation amount and nitrogen reduction condition (W3F3), STI significantly increased (*p*< 0.05) jujube yield compared with CK and achieved the highest net income in both years. Therefore, the W3F3 treatment can be preliminarily recommended as a high−yield and high−efficiency cultivation regime for jujube. However, although there may be room for further increases in yields and economic benefits from increased irrigation, this is not realistic in regions with extreme water scarcity. Instead, it may lead to a reduction in net benefits due to strict government control of irrigation amounts, higher water prices, and the market conditions for jujubes.

**Table 5:** Analysis of economic benefits (¥ ha^−1^) under two irrigation methods in 2021 and 2022.

Treatment	Total input (¥ ha^−1^)		Total output (¥ ha^−1^)		Net income (¥ ha^−1^)		Average net income (¥ ha^−1^)
	2021	2022	2021	2022	2021	2022	
W1F1	17321	14321	47244	43479	29923	29158	29541
W1F2	18626	15626	50546	49668	31920	34042	32981
W1F3	19496	16496	54483	54931	34987	38436	36712
W1F4	19931	16931	45339	43176	25408	26245	25827
W2F1	17526	14526	50673	57961	33147	43435	38291
W2F2	18831	15831	57404	60521	38573	44690	41632
W2F3	19701	16701	60833	66537	41132	49836	45484
W2F4	20136	17136	53340	63223	33204	46087	39646
W3F1	17731	14731	58547	62578	40816	47847	44332
W3F2	19036	16036	68834	77065	49798	61028	55413
W3F3	19906	16906	83312	87660	63406	70754	67080
W3F4	20341	17341	64516	67850	44175	50508	47342
CK	18841	17341	47752	50993	28911	33652	31282

### 3.5 Effect of water and nitrogen regulation on yield and net income

A binary quadratic regression equation (*z*=*ax*^2^+*by*^2^+*cxy*+*dx*+*ey*+*f)* was constructed using the least squares method with irrigation amount *x* (2400–3600 m^3^ ha^−1^), nitrogen amount *y* (286–476 kg ha^−1^) as the independent variable, and *z* as the dependent variable (yield, net benefit).Where *a*, *b*, *c*, *d*, *e* are parameters and *f* is the constant term of the regression equation. Based on the properties of extreme solutions of a binary quadratic regression equation and the range of values of the independent variables *x* and *y*, the regression equation has a maximum value when *x* takes the maximum value. The value of *y* is determined by putting the derivative of *x* into the regression equation.

From the above calculations, it can be concluded that the independent variables *x* and *y* achieve maximum yield of 11795.45 kg ha^-1^ (2021) and 12604.86 kg ha^-1^ (2022) at the boundaries of (3600, 373.97) and (3600, 365.12), respectively. The independent variables *x* and *y* achieved maximum net income of 58,873 ¥ ha^-1^ (2021), 67,233 ¥ ha^-1^ (2022) at the boundaries of (3600, 367.56) and (3600, 367.64). Combining the measured data and regression equation analysis, the irrigation and fertilizer amount were taken as 3600 m^3^ ha^-1^ and 370 kg ha^-1^, respectively, which were more optimal for irrigation and nitrogen application (**Table 6**).

**Table 6:** Regression equations of yield (kg·ha^-1^) and net income (¥·ha^-1^) on irrigation and nitrogen in 2021 and 2022.

Year	Output variable	Regression equation	Irrigation amount (m^3^ ha^-1^)	Nitrogen amount (kg ha^-1^)	Maximum yield (kg ha^-1^) & net income (¥ ha^-1^)
2021	Yield	z = 0.002*x*^2^ − 0.158*y*^2^ + 0.001*xy* − 7.413*x* + 114.561*y* − 4625.750, r^2^ = 0.950*	3600	373.97	11795.45
	Net income	z = 0.011*x*^2^ − 1.028*y*^2^ + 0.004*xy* − 49.731*x* + 741.308*y* − 44025.556, r^2^ = 0.950*	3600	367.56	58873
2022	Yield	z = −1.469×10^-5^*x*^2^ − 0.156*y*^2^ − 0.002*xy* + 4.302*x* + 121.117*y* − 23712.823, r^2^ = 0.949*	3600	365.12	12604.86
	Net income	z = −9.861×10^-5^*x*^2^ − 1.019*y*^2^ − 0.012*xy* + 28.343*x* + 782.457*y* − 167701.334, r^2^ = 0.949*	3600	367.64	67233

* indicates significance at the 0.05 level.

### 3.6 Determination of the optimal irrigation and fertilization schedule

As can be seen from Equations 7–12; **Table 7**, in 2021, the evaluation indicator with the highest weight is NPFP, followed by total outputs and the smallest is total organic acids; and in 2022, the indicator with the highest weight is total inputs, followed by NPFP, and the lowest is V_C_. In both years, the smallest weight of the evaluation indicators was the jujube fruit quality.

**Table 7:** Weights of the evaluation indicators.

Evaluation indicator		Y	WUE	IWUE	NPFP	V_C_	Total output	Net income	Total organic acid	Total input
Weight (%)	2021	15.01	7.52	6.32	15.22	9.06	15.08	13.80	5.77	12.24
	2022	12.59	7.17	6.94	13.45	6.62	12.63	12.25	8.41	19.95

For the relative closeness to the ideal solution *C_i_* (Equations 13–18), the maximum values in both years are W3F2 and W3F1, followed by treatments W3F1 and W3F2, respectively (**Table 8**). According to the rank, the two optimal solutions for 2021 are W3F2 and W3F1, and the two for 2022 are W3F1 and W3F2. The two least optimal solutions for 2021 are CK and W1F4, and the two for 2022 are W1F4 and CK.

**Table 8:** Determination of the optimal water-nitrogen schedule by entropy weighted TOPSIS method.

Treatment	2021				2022			
	$D_{i}^{+}$	$D_{i}^{-}$	*C_i_*	Rank	$D_{i}^{+}$	$D_{i}^{-}$	*C_i_*	Rank
W1F2	0.63	0.53	0.46	6	0.60	0.58	0.49	5
W1F3	0.61	0.53	0.47	5	0.62	0.46	0.43	8
W1F4	0.87	0.25	0.22	10	0.83	0.32	0.28	9
W2F2	0.52	0.56	0.52	3	0.46	0.64	0.58	3
W2F3	0.55	0.49	0.47	4	0.49	0.54	0.52	4
W2F4	0.79	0.30	0.27	8	0.61	0.49	0.45	6
W3F2	0.32	0.73	0.70	2	0.22	0.81	0.79	1
W3F3	0.29	0.88	0.75	1	0.38	0.83	0.68	2
W3F4	0.63	0.43	0.40	7	0.63	0.47	0.43	7
CK	0.88	0.32	0.26	9	0.93	0.11	0.11	10

### 3.7 Correlation analysis of indicators for 2021 and 2022

**Figure 8:**
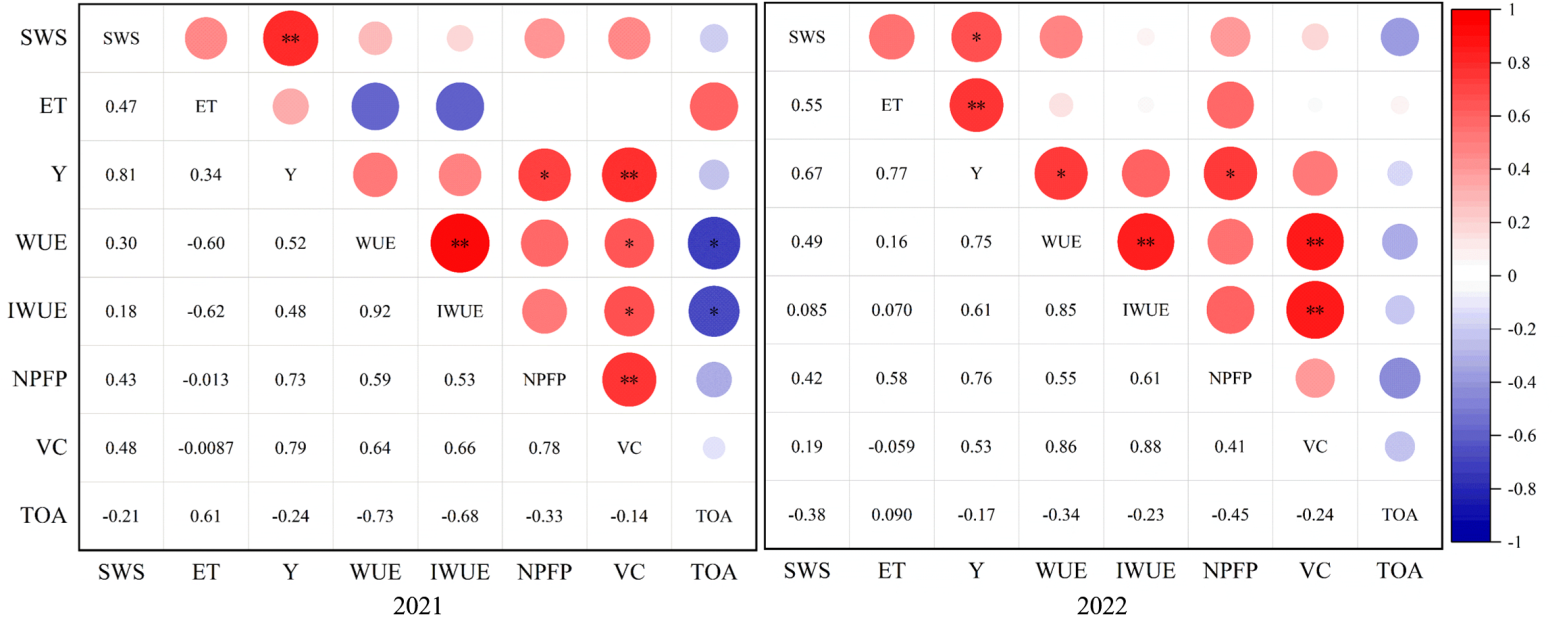
Correlation analysis of indicators for 2021 and 2022.

The correlation analysis of each indicator for 2021 and 2022 is shown in **Figure 8**. As shown in the figure, average SWS and jujube yield exhibit a highly significant (*p*< 0.01, 2021) or significant (*p*< 0.05, 2022) positive correlation. In 2022, ET and yield exhibited a highly significant (*p*< 0.01) positive correlation, whereas in 2021, the correlation was not significant. Over the two years, both yield and NPFP have shown a significant (*p*< 0.05) positive correlation. Additionally, for jujube fruit quality, improved WUE contributes to increased vitamin C content (*p*< 0.05 in 2021, *p*< 0.01 in 2022) and reduced organic acid levels (*p*< 0.05, 2021).

## 4 Discussion

### 4.1 Effects of two irrigation methods on SWS

In this study, irrigation amount very significantly (*p*< 0.01) affected average SWS and it tended to increase with increasing irrigation under STI (**Figure 4**). SWS was significantly higher in STI than in surface drip irrigation under the same irrigation amount. A comparative experiment on soil moisture dissipation found that surface drip irrigation evaporated approximately 30% of the irrigation amount while STI evaporated approximately 4% after 4 days of irrigation (Meshkat et al., 1998). A study on shrubs in a semi-arid zone found that infiltration hole installation significantly increased mean soil water content and reduced soil desiccation (Wang et al., 2020), similar to the water regulation mechanism of STI. STI transfers this water (or puddled water) which used to wet the surface soil to the lower soil layer, which is the main reason for the increase in SWS compared with surface drip irrigation. It is worth mentioning that long-term drying of the surface soil layer may have negative effects on the environment. As Fu et al. (2022) have argued, long-term water-saving irrigation techniques in arid zones have led to an elevated surface temperature and reduced humidity, resulting in a warming-drying effect on the climate. In fact, there is still a great deal of surface wet irrigation in local agricultural production, such as border irrigation for alfalfa and wheat, surface drip irrigation for corn, and sprinkler irrigation for sunflowers. This will, to some extent, lead to a dynamic equilibrium of near-surface temperature and humidity variations, which will in turn weaken the negative environmental effects.

While STI significantly enhanced SWS and crop yield in this study, its long-term effects on soil salinity and nutrient dynamics deserve consideration. In arid regions, the localized water supply and reduced deep percolation under STI may limit salt leaching, potentially leading to gradual salt accumulation in the root zone, especially when irrigation water contains salts or evapotranspiration exceeds the leaching fraction. Changes in soil water content can affect nutrient distribution (Matteau et al., 2022). Therefore, although STI offers short-term benefits for water storage and yield, sustainable application will require periodic monitoring of soil salinity and nutrient status, as well as adaptive management practices such as occasional leaching irrigation or blended fertilizer strategies. Future studies should explicitly address these long-term biogeochemical responses to validate the feasibility of STI in different climatic and edaphic contexts.

### 4.2 Effects of two irrigation methods and nitrogen regulation on ET_a_, WUE, jujube yield, fruit quality, and NPFP

Soil moisture deficit is an important factor affecting crop yield (Kourgialas et al., 2019). Sun et al. (2018) showed that the increase in SWS is a direct factor in the increase of crop yield. In this study, STI increased SWS compared with surface drip irrigation, which is an important reason for its increased yield. Crop evapotranspiration (ET_a_) is closely related to irrigation patterns and crop growth status. Differences in the two irrigation patterns lead to variations in surface evaporation and soil moisture redistribution. Suitable soil hydrothermal and fertilizer conditions are essential for achieving better crop growth and increased yields (Agele et al., 2008; Cao et al., 2023; Melo et al., 2018).

In this study, irrigation was very significant in affecting ET_a_, and it showed a tendency to increase with the increase in irrigation (**Table 3**). WUE tended to decrease and then increase with increasing irrigation amount under STI. Conversely, WUE showed a unimodal trend with increasing irrigation amount under drip irrigation (El‐Hendawy et al., 2008; Kumar Jha et al., 2019). This may be related to the irrigation method and crop species. This study also showed that under the same irrigation amount, STI significantly increases both WUE and IWUE of jujube trees compared with surface drip irrigation. This finding is consistent with the conclusions of Bai et al. (2022); Sun et al. (2016) on jujube trees, mainly because sand-tube irrigation significantly increases soil water storage and reduces surface evaporation. The highest WUE was obtained in 2021 under low water and medium nitrogen, while in 2022 it was obtained under high water and medium nitrogen. The maximum IWUE and yield were obtained in two years under high water and medium nitrogen conditions. This suggests that there is uncertainty in the effect of irrigation amount on WUE. Perhaps, for example, less irrigation may result in the root system of jujube trees ingesting deep soil moisture to satisfy their water needs (Ma et al., 2013, 2012). However, it also suggests that appropriate nitrogen amounts are one of the most essential approach to increase WUE. Maximum IWUE and jujube yield were obtained with maximum irrigation amount and medium nitrogen (W3F3) under STI (**Table 3**). This shows that high water and medium nitrogen with STI is the preferred option for improving yields and IWUE, and is one of the most important pathways to solving water scarcity.

In this study, the maximum irrigation amount under STI was the same as that of conventional surface drip irrigation, yet the former had a significantly higher yield than the latter. This further indicates that the STI technique for cultivating jujube trees is a key technology for water saving and yield enhancement. A key factor contributing to the yield increase is the significant enhancement of soil water storage (**Figure 4**), which in turn created a favorable water environment. Furthermore, nitrogen levels very significantly affected yield in both years, and the jujube yield exhibited a unimodal trend with increasing nitrogen (**Table 3**). This is in line with the results of Dai et al. (2019) on jujube trees.

### 4.3 Effects of two irrigation methods and nitrogen regulation on fruit quality

Vc is one of the most important indicators of jujube fruit quality. In this study, the treatments with the highest Vc content were W1F1 (2021) and W3F3 (2022), respectively. This suggests that inadequate irrigation reduces water productivity but may enhance fruit quality. The flowering and fruit setting rate of jujube trees was greatly affected when the amount of water and nitrogen applied was reduced, which eventually led to a decrease in the number of fruit set. Conversely, due to less fruit setting, better quality was obtained with certain water and nitrogen supply. In addition, appropriate water and nitrogen supply (high water and medium nitrogen, W3F3) improves jujube yield and quality. However, improved yield is not necessarily an improvement in quality. This is not consistent with the results of Wang et al. (2024) who concluded that subsurface drip irrigation increased crop yield but failed to improve quality, likely because high water and nitrogen inputs promote vegetative growth at the expense of fruit quality. From the overall analysis, high water and nitrogen favored improvement all quality indicators, but the differences between some treatments were not significant, which may be related to the genetic characteristics of jujube trees. Liu et al. (2014) concluded that the high vitamin C content of jujube can be attributed to the unique high level of expression of genes involved in biosynthesis and regeneration, which supports the present study on jujube fruit quality. Consequently, even though high water and nitrogen inputs favored yield improvement and showed positive trends in some quality indicators of jujube, the inherent genetic ceiling of jujube limits the magnitude of treatment differences, making them non-significant in certain comparisons.

### 4.4 Optimal irrigation and nitrogen strategies based on TOPSIS method

The TOPSIS method offers several advantages for the comprehensive evaluation of irrigation and nitrogen management schemes. In this study, the entropy weight method was used to assign objective weights to each indicator, thereby avoiding the bias associated with subjective weighting by researchers. However, the entropy-weighted TOPSIS method has inherent limitations. The selection of evaluation indicators and their normalization methods still involves subjective decisions by the researcher, which may affect the results. Furthermore, the entropy weights are dependent on the current dataset; adding or removing treatments or indicators could alter the weight distribution. The optimal water and nitrogen management strategy should take into account jujube yield, water and nitrogen use efficiency, fruit quality, total input, total output, and net income. Higher yields are associated with increased irrigation, which can lead to lower WUE as well as an increase in total inputs (**Table 3**). Also, higher yields do not necessarily mean better quality. In this study, maximum yield and highest net income were obtained for high water and medium nitrogen (W3F3). According to the TOPSIS method, in 2021, the 1st and 2nd ranked treatments were W3F3 and W3F2, respectively, and in 2022, the 1st and 2nd ranked treatments were W3F2 and W3F3, respectively. Therefore, due to its high yield and high net income, W3F3 was determined to be the optimal water and nitrogen management solution based on the TOPSIS method.

## 5 Conclusion

Sand tube irrigation (STI) combined with high water and medium nitrogen significantly increased soil water storage, jujube yield, water and nitrogen use efficiency. Increasing irrigation amount under STI has a greater room for improving jujube yield and net income. However, due to water scarcity constraints and based on irrigation experience, there is no need to increase the irrigation amount further. Using the binary quadratic regression equation and the TOPSIS method, the optimal water and nitrogen amounts for STI in jujube tree were determined to be 3600 m^3^ ha^−1^ and 370 kg ha^−1^, respectively. Our findings provide a new perspective and theoretical basis for the application of new technologies and efficient management of water and nitrogen for jujube trees in arid regions. The research may also provide methodological guidance for water-saving cultivation of crops in arid regions around the world. It is worth noting that the STI technique and nitrogen amount in this study were limited by the jujube trees’ age and size, the soil characteristics, local water resources and climate. In addition, the irrigation amount was the same in all growth periods in this study. Further research will be conducted to investigate the effect of water and nitrogen coupling on the jujube tree’s water saving and yield increasing under different irrigation amounts in each growth period, as well as the long−term impacts on soil salinity and nutrient distribution, and the applicability of STI to other jujube varieties.

## Data Availability

The original contributions presented in the study are included in the article/supplementary material. Further inquiries can be directed to the corresponding author.
